# MDTR: a knowledge-guided interpretable representation for quantifying liver toxicity at transcriptomic level

**DOI:** 10.3389/fphar.2024.1398370

**Published:** 2025-01-24

**Authors:** Inyoung Sung, Sangseon Lee, Dongmin Bang, Jungseob Yi, Sunho Lee, Sun Kim

**Affiliations:** ^1^ Interdisciplinary Program in Bioinformatics, Seoul National University, Seoul, Republic of Korea; ^2^ Institute of Computer Technology, Seoul National University, Seoul, Republic of Korea; ^3^ AIGENDRUG Co., Ltd., Seoul, Republic of Korea; ^4^ Interdisciplinary Program in Artificial Intelligence, Seoul National University, Seoul, Republic of Korea; ^5^ Department of Computer Science and Engineering, Seoul National University, Seoul, Republic of Korea

**Keywords:** drug-induced liver injury, one-class boundary, kernel distance, transcriptomic signature, degree of toxicity, liver toxicity

## Abstract

**Introduction:**

Drug-induced liver injury (DILI) has been investigated at the patient level. Analysis of gene perturbation at the cellular level can help better characterize biological mechanisms of hepatotoxicity. Despite accumulating drug-induced transcriptome data such as LINCS, analyzing such transcriptome data upon drug treatment is a challenging task because the perturbation of expression is dose and time dependent. In addition, the mechanisms of drug toxicity are known only as literature information, not in a computable form.

**Methods:**

To address these challenges, we propose a Multi-Dimensional Transcriptomic Ruler (MDTR) that quantifies the degree of DILI at the transcriptome level. To translate transcriptome data to toxicity-related mechanisms, MDTR incorporates KEGG pathways as representatives of mechanisms, mapping transcriptome data to biological pathways and subsequently aggregating them for each of the five hepatotoxicity mechanisms. Given that a single mechanism involves multiple pathways, MDTR measures pathway-level perturbation by constructing a radial basis kernel-based toxicity space and measuring the Mahalanobis distance in the transcriptomic kernel space. Representing each mechanism as a dimension, MDTR is visualized in a radar chart, enabling an effective visual presentation of hepatotoxicity at transcriptomic level.

**Results and Discussion:**

In experiments with the LINCS dataset, we show that MDTR outperforms existing methods for measuring the distance of transcriptome data when describing for dose-dependent drug perturbations. In addition, MDTR shows interpretability at the level of DILI mechanisms in terms of the distance, i.e., in a metric space. Furthermore, we provided a user-friendly and freely accessible website (http://biohealth.snu.ac.kr/software/MDTR), enabling users to easily measure DILI in drug-induced transcriptome data.

## 1 Introduction

Drug-induced hepatotoxicity (also known as drug-induced liver injury; DILI) is a serious issue for both drug development and patient safety (Kaplowitz, 2004; Wilke et al., 2007). Traditional studies of toxicity have used animal models. This approach is time-consuming and costly, and may not accurately predict human toxicity (Van Norman, 2019; Bracken, 2009). Meanwhile, *in vitro* bioassays offer a more direct insight into human biology, with lower costs and ethical concerns (Chapman et al., 2013; Van Norman, 2019). Because these bioassays enable high-throughput screening, there is a growing interest to use the bioassays for toxicity signature screening to analyze toxicity at the individual patient cases.

The increasing availability of large-scale chemical libraries and gene expression data has significantly advanced our ability to investigate drug-induced toxicities. Several public resources have been developed to facilitate high-throughput screening and toxicity profiling. Among these, Tox21, a collaborative US federal research program, focuses on developing *in vitro* assays to screen for potentially toxic chemicals. Tox21 has pioneered the use of medium-to high-throughput panel assays to test thousands of chemicals for potential toxicity (Andersen and Krewski, 2009; Krewski et al., 2010). ToxCast, another program led by the US Environmental Protection Agency, extends the capabilities of Tox21 by offering medium- and high-throughput screening data for a wide range of chemicals (Dix et al., 2007). However, high throughput panel assays cannot measure toxic effect of drug at the transcriptomic level, thus molecular level mechanism of action (MoA) of drug response cannot be analyzed. To overcome the limitation of the panel assays, other resources such as the Genomics of Drug Sensitivity in Cancer (GDSC, Garnett et al. (2012)) and the Cancer Cell Line Encyclopedia (CCLE, Barretina et al. (2012)) provide valuable gene expression data and drug response metrics, such as IC50 and AUC values, across various cancer cell lines. However, both GDSC and CCLE lack the gene expression data that reflect post-drug treatment states. To address these gaps, the Library of Integrated Network-Based Cellular Signatures (LINCS) serves as a comprehensive resource that provides *in vitro* gene expression data after drug treatment (Subramanian et al., 2017). LINCS allows for the analysis of drug-induced perturbations over time and across different doses in various cell lines.

### 1.1 Challenges

Understanding how a drug interacts and affects biological systems to induce hepatotoxicity with the drug-induced transcriptome data is a goal of this study. The perturbed biological mechanisms by dysregulated genes can be interpreted as a degree of hepatotoxicity at transcriptomic level. However, despite the abundance of drug response and gene expression data, measuring the degree of drug-induced hepatotoxicity faces three major challenges.

1. Data availability varies greatly depending on drug treatment conditions. For example, the LINCS dataset includes gene expression values for 12,328 genes across an average of 16 samples (with a standard deviation of 30) for varying dose and time point combinations (Supplementary Figure S1). It causes a high-dimensional low-sample issue and hinders accurate interpretation of the MoA of drugs.
2. Identifying toxic patterns or signatures among drug-treated expression data is challenging. In the current state of knowledge, hepatotoxic signatures at transcriptomic level are insufficient (Andrade et al., 2019), and these signatures are dose- and time-dependent, as exemplified by hormesis (Mattson, 2008). Furthermore, biological mechanisms related to hepatotoxicity are available only in the literature. If possible, we need computational method to translate drug-induced transcriptome data to known biological mechanisms related to hepatotoxicity.
3. Defining the boundaries of toxicity is complex. Cell survival is governed by maintaining homeostasis, which is influenced by various conditions such as temperature and oxygen levels (Chovatiya and Medzhitov, 2014). Any disruption beyond the homeostasis boundary can lead to cell death with cytotoxic effects. Consequently, the state of toxicity cannot be confined within a specific boundary between toxic and non-toxic labels.

### 1.2 Our approach

To address these challenges, we propose MDTR, a knowledge-guided Multi-Dimensional Transcriptomic Ruler for quantifying the degree of drug-induced hepatotoxicity at the transcriptomic level. We first compiled five biological mechanisms of hepatotoxicity from the recent literature (Andrade et al., 2019; Weaver et al., 2020; Han et al., 2020). To translate transcriptome data to the five hepatotoxicity mechanisms, MDTR incorporates KEGG pathways as representative of mechanisms, mapping transcriptome data to these pathways and subsequently aggregating them for each of the five hepatotoxicity mechanisms. This involves three steps: (1) Identifying the most-perturbed transcriptomic samples as outliers using Dual-SVDD. The rationale behind this assumption is that toxic signatures are distinct from all remaining transcriptomic samples. (2) Constructing a transcriptomic embedding space using the Radial Basis Function (RBF) kernel and defining a ruler in the kernel space. (3) Extending the ruler into a five-dimensional radar chart for interpreting hepatotoxicity based on the knowledge-guided biological mechanisms.

As a result, MDTR represents *the degree of DILI as the distances* that measure the perturbation of the biological mechanisms under the drug treated environment. MDTR distance outperforms existing methods, which measure distances between transcriptomic samples, in reflecting the dose-dependent effects of drug on liver injury. In addition to its quantitative capabilities, MDTR provides interpretive power for understanding the MoA. We also provide a website for calculating and visualizing the five-dimension radar chart using drug-treated transcriptomic data input by scientists.

## 2 Methods and materials

**FIGURE 1:**
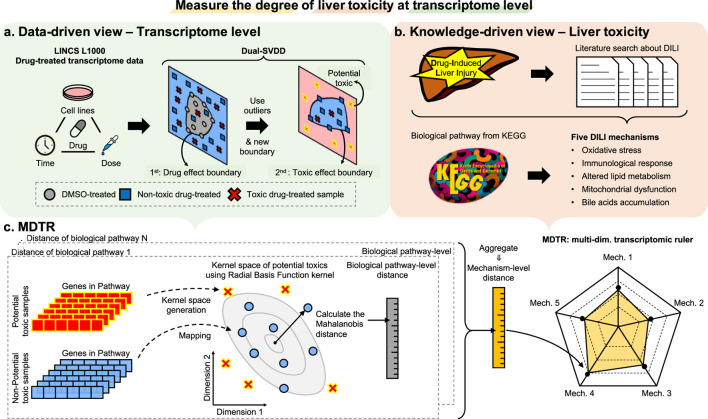
The overview of MDTR. To measure the degree of liver toxicity at the transcriptome level, hepatotoxic signatures were explored through both **(A)** data- and **(B)** knowledge-driven views, and **(C)** a knowledge-guided multi-dimensional ruler MDTR was proposed. **(A)** Dual-SVDD workflow. For drug-treated transcriptome data of LINCS, Dual-SVDD generates dual-boundaries to identify the most perturbed, i.e., potential toxic samples. **(B)** Literature search workflow. Five major DILI mechanisms were proposed through literature searches, and the corresponding biological pathways were collected from KEGG. **(C)** MDTR workflow. Each biological pathway within a mechanism calculates a pathway-level distance in the Radial Basis Function (RBF) kernel space. These pathway-level distances are then aggregated to form the mechanism-level distance, which serves as an axis in the multi-dimensional ruler.

In this section, we introduce the details of MDTR, which aims to measure the degree of hepatotoxicity from drug-induced transcriptome data. MDTR consists of two steps: (1) Exploration of hepatotoxic signatures through data- and knowledge-driven view. (2) Calculation of the degree of hepatotoxicity by a knowledge-guided multi-dimensional ruler. Figure 1 illustrates the overview of MDTR.

### 2.1 Exploration of hepatotoxic signatures at transcriptomic level

As mentioned in challenge #2, the signatures that can be utilized to measure drug-induced hepatotoxicity at the cellular level have not yet been clearly defined. To bridge the current gap in available data and knowledge, we explore hepatotoxic signatures through two approaches: (i) data-driven view: identification of potential toxic samples by Dual-SVDD. (ii) knowledge-driven view: literature mining of biological mechanisms related to drug-induced hepatotoxicity.

#### 2.1.1 Data-driven view: identification of potential toxic samples by Dual-SVDD

Upon drug treatment, biological mechanisms are perturbed, leading to changes in gene expression levels. As mentioned in challenge #2, although the toxic signatures may not be clear, samples that are significantly influenced by the toxic drug, either by the treated time or dose, may show significant dysregulation in gene expression. Indeed, the perturbation signature of LINCS is known to be associated with cell viability linked to regulation of transcription factors such as apoptosis and proliferation (Szalai et al., 2019). In particular, toxic compounds can induce cell death signatures, suggesting the potential to predict MoA through drug-specific perturbation profiles (Niepel et al., 2017). Based on this understanding, we assumed that samples containing a toxic signature would show distinct perturbations among the toxic-treated transcriptome data, and we referred to these samples as “potentially toxic (PT)” samples. However, in the process of selecting PT samples, as mentioned in challenge #3, the processes of dysregulation due to toxicity are diverse, making it difficult to define them with a single, closed boundary. Therefore, we designed an approach of defining boundaries from relatively homogeneous effects and detecting PT samples as outliers compared to other drug-induced transcriptome samples.

To identify the PT samples, we propose a novel method called Dual-SVDD, which consists of two boundaries: the drug-effect boundary and the toxic-effect boundary. This method is based on the construction of a one-class boundary method, Support Vector Data Description (SVDD, Tax and Duin (2004)). Formally, given a set of samples $\left(x_{i},y_{i}\right)$ where $x_{i}$ is a gene expression profile measured after treatment of drugs or dimethyl sulfoxide (DMSO), and $y_{i}$ is 1 if $x_{i}$ is a DMSO-treated sample, and −1 otherwise (for toxic or non-toxic drug-treated samples), the drug-effect boundary is a hypersphere that encloses a majority of the DMSO-treated samples while minimizing distance between the center of the sphere and the closest DMSO-treated samples on the boundary. To address the non-linearity of gene interactions, we employed the RBF kernel in the optimization of the hypersphere using SVDD:

$$\begin{array}{cc} \underset{r,b,\alpha }{\min}\; & r^{2}+\frac{1}{n}\overset{n}{\underset{i=1}{\sum }}\alpha_{i}\\ \;s.t.\;y_{i} & \left(\overset{n}{\underset{j=1}{\sum }}\alpha_{j}k\left(x_{j},x_{i}\right)+b-r^{2}\right)\leq 1,\;\alpha_{i}\geq 0,\;\forall i \end{array}$$ (1)

where r is the radius of the sphere, n is the number of samples, $\alpha_{i}$ is a Lagrange multiplier, b is a bias term, $k\left(x_{j},x_{i}\right)=\exp\left(-\gamma |x_{i}-x_{j}{|}^{2}\right)$ is the RBF kernel function with width control parameter γ . In this study, γ is empirically set to 1/(d∗σ) where d is the number of genes in $\left\{x_{i}\right\}$ and σ is the variance of $\left\{x_{i}\right\}$ .

The samples with $\alpha_{i}>0$ constitute the support vectors of the drug-effect boundary. Using the support vectors, for a new sample x , the decision function $f^{DE}\left(x\right)$ is defined as below.

$$f^{DE}\left(x\right)=\overset{n}{\underset{j=1}{\sum }}\alpha_{j}k\left(x_{j},x\right)+b-r^{2}$$ (2)

when $f^{DE}\left(x\right)\leq 0$ , x is classified as a sample without drug-effect; otherwise, it is classified as a sample with drug-effect.

Then, we additionally designed a toxic-effect boundary. Instead of DMSO-treated samples, we utilized a target dataset $X^{NT}$ consisting of non-toxic drug-treated samples where $f^{DE}\left(x_{i}^{NT}\right)>0$ . A test dataset $X^{T}$ was also constituted using toxic drug-treated samples where $f^{DE}\left(x_{i}^{T}\right)>0$ .

Using the $X^{NT}$ dataset and Equations 1, 2, we obtained the toxic-effect boundary and the decision function $f^{TE}\left(x\right)$ . The dual-boundaries generated by Dual-SVDD are represented by $f^{DE}\left(x\right)=0$ and $f^{TE}\left(x\right)=0$ , which are used to identify samples with potential drug effects and potential toxic signatures, respectively. We refer to samples in $X^{PT}=\left\{x_{1}^{PT},\ldots ,x_{l}^{PT}\right\}$ as ‘potentially toxic (PT)’ samples, where $x_{i}^{PT}\in X^{T}$ and $f^{TE}\left(x_{i}^{PT}\right)>0$ . These PT samples are considered to have a higher likelihood of possessing toxic signatures.

#### 2.1.2 Knowledge-driven view: biological mechanisms related to drug-induced liver injury

Through a comprehensive literature review, we curated commonly discussed biological mechanisms related to hepatotoxicity in existing studies (Andrade et al., 2019; Weaver et al., 2020; Han et al., 2020), and summarized them into five biological mechanisms: *Oxidative stress*, *Immunological response*, *Altered lipid metabolism*, *Mitochondrial dysfunction*, and *Bile acids accumulation* (Table 1).

**TABLE 1:** The five hepatotoxicity mechanisms used in the MDTR. The table shows the mechanisms of hepatotoxicity categorized into five major groups based on literature search, serving as the axis of MDTR. For each mechanisms, it shows the action in the liver and the number of KEGG pathways belonging to the mechanism.

Mechanism	Hepatotoxicity process	Num. of pathways
Oxidative stress	Disruption of essential molecules through production of ROS	6
Immunological response	Immunological response including inflammation induced by drugs or metabolites	3
Altered lipid metabolism	Disruption of normal lipid metabolism and accumulation of lipids leading to tissue damage	13
Mitochondrial dysfunction	Impairment of MT function and subsequent disruption of cellular energy metabolism	2
Bile acids accumulation	Accumulation of bile acids and impaired bile flow in the liver can cause liver injury	2

*Num.: Number, ROS: Reactive oxygen species, MT: Mitochondria.

These biological mechanisms are conceptually associated with hepatotoxicity but their applicability in a computational approach is not known. To address this, we explored biological pathways associated with the mechanisms and utilized the gene sets within those pathways. To identify pathways relevant to these mechanisms, we used the KEGG Pathway Search function, which conducts a keyword search against the KEGG pathway database (Kanehisa, 2002). As the search function performs partial matches on text descriptions or legends for multiple keywords, there is a possibility of false positives in the search results. For example, when “oxidative stress” is searched, the term ‘stress’ may detect other stress-related pathways. Moreover, even if keywords are included in the description, there may be portions that are far from the main function of the pathway. Thus, we manually curated the search results to ensure their relevance to the mechanism. Further details of the selection process for each mechanism and the selected pathway list are provided in Supplementary Methods section and Supplementary Tables S1-S5.

### 2.2 Calculation of the degree of hepatotoxicity by a knowledge-guided multi-dimensional ruler

Through the exploration of hepatotoxic signatures from both data-driven and knowledge-driven perspectives, we identified PT samples with a high likelihood of harboring drug-induced hepatotoxic signatures, as well as biological mechanisms associated with hepatotoxicity and corresponding pathways. Leveraging this valuable information, we introduced MDTR, quantifies the degree of hepatotoxicity as dysfunctions of biological mechanisms. The MDTR is represented as a five-dimensional radar chart, with each dimension corresponding to a biological mechanism, calculated through *mechanism-level toxic distances*.

Formally, given a biological mechanism $M=\left\{p_{1},\ldots ,p_{k}\right\}$ of k pathways, the mechanism-level toxic distance $D_{M}\left(x\right)$ (Equation 3) is defined as a sum of multiple weighted *pathway-level distances* as below:

$$D_{M}\left(x\right)=\underset{p\in M}{\sum }w\left(p,x\right)d_{p}\left(x\right)$$ (3)

where w(p,x) (Equation 5) is a weight of pathway p with respect to x and $d_{p}\left(x\right)$ (Equation 4) is a pathway-level distance that measures the activity of how the pathway p is dysregulated on x , which is calculated on the non-linear kernel space to learn complex interactions of genes.

Given a pathway $p=\left\{g_{1},\ldots ,g_{l_{p}}\right\}$ consisting of $l_{p}$ genes and PT samples $X_{p}^{PT}$ , the toxicity kernel space $H_{p}\in R^{d}$ is derived from $X_{p}^{PT}$ using KernelPCA with RBF kernel, implemented via the Python Scikit-learn package. The gamma parameter γ is set to 1/l , while all other parameters remain at their default settings. To construct the toxicity kernel space $H_{p}$ , the dimensionality d of $H_{p}$ is determined by the first $\min\left(0.1*l_{p},3\right)$ principal components. By considering the relationships between transcriptomic samples in the latent space represented by genes within pathways, we can alleviate the high dimensionality issue mentioned in challenge #1. After the construction of toxicity kernel space, PT samples $X_{p}^{PT}$ and non-PT samples $X_{p}^{\mathrm{non-PT}}$ are mapped into the toxicity kernel space $H_{p}$ with sample distribution $Q_{p}\in R^{d}$ . Then, a pathway-level distance $d_{p}\left(x\right)$ is calculated as the Mahalanobis distance in the toxicity kernel space of the mapped samples:

$$d_{p}\left(x\right)=\sqrt{{\left(z-\mu_{p}\right)}^{T}S_{p}^{-1}\left(z-\mu_{p}\right)}$$ (4)

where z represents the latent embedding of the sample x mapped into the kernel space $H_{p}$ . $\mu_{p}$ and $S_{p}$ are the mean vector and the positive-definite covariance matrix derived from $Q_{p}$ , respectively. In other words, once the toxicity kernel space is constructed using PT samples, all samples, including both PT and non-PT samples, are mapped into this space. The Mahalanobis distance is then calculated for each sample in this toxicity kernel space. After calculating $d_{p}\left(x\right)$ for all samples, min-max normalization is performed to adjust the scale.

The weight w(p,x) represents the significance of dysregulation of the pathway p in the sample x against the pathway-level distances of $X_{p}^{\mathrm{non-PT}}$ . Formally, as our observation, the distribution of $\left\{d_{p}\left(x\right)|x\in X_{p}^{\mathrm{non-PT}}\right\}$ follows F-distribution. Then, the weight w(p,x) is calculated as:

$$\begin{array}{ll} w\left(p,x\right) & =-\log\left(F\left(d_{p};d_{1},d_{2}\right)\right)\\ & =-\log\left(I_{d_{1}\cdot d_{p}/\left(d_{1}\cdot d_{p}+d_{2}\right)}\left(\frac{d_{1}}{2},\frac{d_{2}}{2}\right)\right) \end{array}$$ (5)

where $d_{p}$ denotes $d_{p}\left(x\right)$ . $d_{1}$ and $d_{2}$ are degrees of freedom that measured from $\left\{d_{p}\left(x\right)|x\in X_{p}^{\mathrm{non-PT}}\right\}$ . I is the regularized incomplete beta function.

### 2.3 Website for measuring the degree of toxicity through MDTR

The website (http://biohealth.snu.ac.kr/software/MDTR) is designed to measure the degree of liver toxicity of drugs by analyzing their gene expression data. The website uses MDTR to show the potential liver toxicity of drugs across the five mechanisms of hepatotoxicity.

Additionally, the website leverages the LINCS dataset to access gene expression data from 514 drugs across 70 different cell lines, specifically utilizing L1000 Level 5 data, which are generated by calculating z-scores relative to the controls. This provides a comprehensive assessment of potential hepatotoxicity at the transcriptomic level. The website is implemented using the Django 3.2 framework and Bootstrap 4.6 for the front-end. The interactive radar chart, representing the indicators of liver toxicity, is generated using Chart. js. Each axis of the radar chart represents an indicator of liver toxicity. The website is tested for compatibility and functionality on various web browsers, including Chrome, Microsoft Edge, Firefox, and Safari.

### 2.4 Preparation of drug hepatotoxicity information and drug-induced transcriptome data

Two databases, Drug Induced Liver Injury Rank (DILIrank, Chen et al. (2016)) and LiverTox (Hoofnagle, 2013), were used to gather information on drug-induced hepatotoxicity. DILIrank categorizes drugs into four classes based on their potential for causing hepatotoxicity, while LiverTox assigns likelihood scores indicating the extent of reported liver injury cases. For this study, drugs categorized as most-DILI concern in DILIrank, and A and B in LiverTox were considered hepatotoxic. Drugs categorized as no-DILI concern in DILIrank, and D and E in LiverTox were considered non-hepatotoxic. A total of 220 hepatotoxic drugs and 402 non-hepatotoxic drugs were used in the analysis.

Drug-treated samples from the LINCS database (accession number GSE92742) were obtained for investigating drug-induced hepatotoxicity at the transcriptomic level. For the gene expression matrix of these samples, we used Level 5 data, which are generated by calculating z-scores relative to the controls. Categorization of these samples into DMSO, non-toxic, and toxic groups was guided by annotations from DILIrank and LiverTox. A total of 20,529 drug-treated samples (6,405 toxic, 11,333 non-toxic and 2,791 DMSO-treated samples) were collected. From the LINCS dataset, among the 10,174 LINCS best inference genes, 4,693 genes related to drug-induced liver injury were selected based on the Comparative Toxicogenomics Database (CTD, Davis et al. (2021)) and enrichment analysis of Gene Ontology (GO) terms. Further description and discussion of drug hepatotoxicity information, transcriptome data collection, and the selection of gene sets are provided in the Supplementary Methods section.

## 3 Results and discussion

### 3.1 MDTR: a knowledge-guided representation for liver toxicity at transcriptomic level

Among the 1,896 drug-treated transcriptome samples, Dual-SVDD selected the most perturbed samples, the extreme point of MDTR. As a result, 64 potentially toxic (PT) samples were identified (Supplementary Table S6). We showed the utility of Dual-SVDD through its ability to distinguish toxic samples and the robustness of dual-boundaries (Supplementary Figure S2). In addition, to evaluate the reliability of the proposed measurement, we conducted self-validation by partitioning the total dataset and performing cross-check analyses (Supplementary Figure S3). As a result, our measurement method showed consistent distances regardless of the configuration of the data.

**FIGURE 2:**
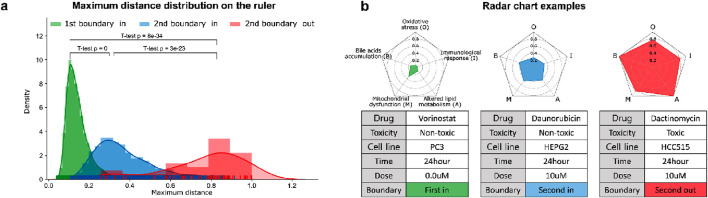
The results of MDTR. **(A)** Maximum distance distribution on the ruler. The density distribution shows the maximum values on the MDTR of the samples divided according to the Dual-SVDD boundary. **(B)** Examples of multi-dimensional rulers using radar charts for drug-treated transcriptome samples.

Based on the PT samples and the curated biological mechanisms, MDTR measures the degree of DILI. Figure 2A shows a distribution of maximum distances, representing the density distribution of maximum values on the MDTR of the samples. Notably, significant statistical differences were observed between the sample groups identified by Dual-SVDD. This indicates that the MDTR distance measured from PT samples meaningfully reflects the perturbation of gene expression affected by drug treatments. Among the drug-induced transcriptome data, Figure 2B illustrates examples of radar charts from MDTR. The red-colored radar chart (Figure 2B, right), representing the treated compound labels as structural toxicity as ‘Toxic’, shows greater distances in comparison to the other two examples. Interestingly, even when the chemical structural information is identical and labeled as ‘Non-toxic’, the MDTR results of the two drug-induced transcriptome samples (green vs. blue-colored) show obvious differences. This indicates the importance of analyzing drug hepatotoxicity at the transcriptome level.

**FIGURE 3:**
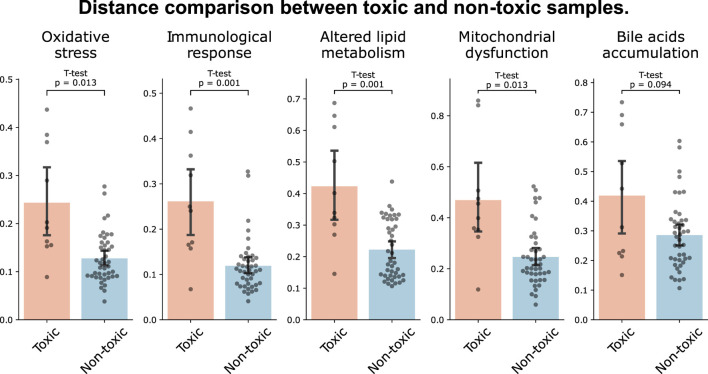
Distance comparison between toxic and non-toxic samples. The bar plot shows the distances between samples of toxic drugs (red) and samples of drugs with unknown toxicity (blue) in liver cell lines.

We further investigated the capability of MDTR in identifying samples treated with hepatotoxic drugs. We screened top-rankded drugs for liver toxicity, labeled as ‘Most-DILI’ in DILIrank or assigned category ‘A’ in LiverTox, as well as drugs with an absence of reported toxicity, labeled as ‘No-DILI’ in DILIrank or category ‘E’ in LiverTox. Then, we used samples treated with the selected drugs in liver cell lines, including HEPG2, HUH7, and PHH. This resulted in the identification of 10 toxic and 43 non-toxic samples, respectively. When comparing the distances between the two sample groups, we observed larger distances for toxic samples across all mechanisms, with statistically significant differences found in all mechanisms except for Bile acids accumulation (Figure 3). Interestingly, similar results were observed when the experiment was conducted not only in liver cell lines but also in the entire cell lines belonging to the data used (Supplementary Figure S4). Therefore, we showed the effectiveness of the multi-dimensional ruler in stratifying toxic and non-toxic samples at the transcriptomic level.

### 3.2 Comparison of dose-dependent liver toxicity

Our research goal is not only to measure the five-dimensional toxic mechanisms in drug-treated transcriptomic samples, but also to facilitate the understanding of toxicity mechanisms using the multi-dimensional ruler in the form of the radar chart. We explored the drug treated environment, especially the sample distance according to the drug dose. For dose-dependence analysis, a total of 198 combinations of 33 drugs with five or more drug dose points in fixed cell line and time point were used. Under the assumption that toxicity increases with increasing drug dose (Schenker et al., 1999; Hoofnagle and Björnsson, 2019), the correlation between dose and distance was calculated, and then evaluated that higher correlation resulted in better results. We compared MDTR distance with three methods: (1) Mathematical distances (Cosine, Euclidean, and Mahalanobis) calculated from the expression values of all genes in LINCS, (2) Transcriptomic Signature Distance (TSD, Manatakis et al. (2020)), and (3) Pathway Activity Score Learning (PASL, Karagiannaki et al. (2023)) with mathematical distances similar to (1) (details in Supplementary Methods).

As shown in Table 2, MDTR outperformed the other distance methods, achieving 70% positive ratio. Additionally, MDTR consistently showed the highest positive correlation ratio even when the number of drug dose points was three or four (Supplementary Table S7). Notably, the Mahalanobis distance yielded better results than Cosine and Euclidean distances. In addition, distances calculated based on biological information (MDTR and PASL) exhibited better performances compared to using the entire genes without the prior knowledge.

**TABLE 2:** Performance comparison for dose-distance relationship. The table shows the ratio (in numbers) of samples exhibiting a positive correlation between dose and distance among the 198 samples for MDTR and three comparison methods. Bold values indicate the best results.

Method	Positive ratio (Num. of positive samples)
MDTR	**0.70 (139)**
Raw + Cosine	0.62 (123)
Raw + Euclidean	0.62 (122)
Raw + Maha	0.64 (127)
TSD	0.64 (127)
PASL + Cosine	0.53 (105)
PASL + Euclidean	0.61 (121)
PASL + Maha	0.67 (133)

*Num.: Number, Maha.: Mahalanobis.

The main advantages of MDTR are that it not only provides distances indicating the degree of toxicity depending on the drug-treated environment but also allows interpretation of the mechanisms of liver toxicity. Among drugs that show a positive correlation with MDTR and dose, we conducted case studies for the following three drugs: Doxorubicin, Mitoxantrone, and Rosiglitazone.

**FIGURE 4:**
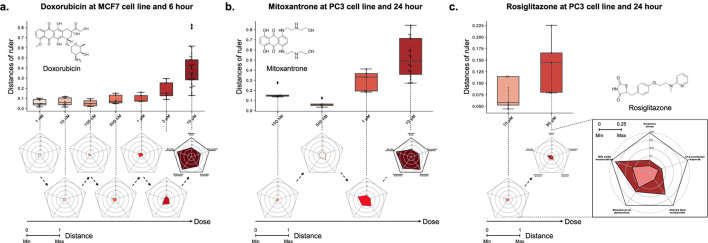
The relationship between the distance on the multi-dimensional ruler and the dose. The box plots and radar charts show the change in distance based on the dose of drug treatment under fixed cell line and time point. **(A)** Doxorubicin at MCF7 cell line and 6-hour, **(B)** Mitoxantrone at PC3 cell line and 24-hour, and **(C)** Rosiglitazone at PC3 cell line and 24-hour. Except the zoomed radar chart, all radar charts exhibit a scale ranging from a minimum of 0 to a maximum of 1.

Case 1 We investigated Doxorubicin, a chemotherapy medication widely used for the cancer treatments, including breast and bladder cancer, while also known for its hepatotoxicity (Prasanna et al., 2020). In the MCF7 cell line and at the 6-h time point, Doxorubicin exhibited dose-dependent toxicity signature (Figure 4, ρ = 0.93, *p* = 1.1e-6). These positive correlations were observed in 9 out of the 10 Doxorubicin-treated combinations (Supplementary Table S8). In particular, the radar chart revealed distinct changes across different dose points in two mechanisms: Mitochondrial dysfunction and Bile acids accumulation. These findings support the understanding of the potential association between bile acid metabolism and Doxorubicin sensitivity (Chen et al., 2017), as well as the preferential accumulation of Doxorubicin in the mitochondria and nucleus (Wallace et al., 2020).

Case 2 We investigated the potential toxicity of Mitoxantrone, a cytotoxic agent and anthracenedione analogue of Doxorubicin (Fox, 2004), which exhibits similar metabolic pathways (Paciucci and Sklarin, 1986; Llesuy and Arnaiz, 1990; Rossato et al., 2014a). Mitoxantrone, despite being classified with a likelihood score of D in the LiverTox database, showed a positive correlation between distance and dose across all combinations of MCF7, PC3, and A549 cell lines and 6 and 24 h time points, similar to Doxorubicin (Supplementary Table S9). For example, Figure 4B illustrates the dose-dependent changes in distance for Mitoxantrone in the PC3 cell line at the 24-hour time point ( ρ = 0.87, *p* = 0.012).

In particular, the toxicity of the sample showed a non-monotonic tendency that decreased in the early stage of dose escalation (from 0.1 to 0.5 μ M) and then increased again after the 1uM dose (Tang et al., 2023). These results showed that MDTR is capable to capture the pattern of drugs showing such hormesis. Overall dose-distance relationship suggest that Mitoxantrone not only induces functional impairments observed with Doxorubicin, such as bile acids accumulation and mitochondrial dysfunction, but also affects lipid metabolism (Kharasch and Novak, 1985; Rossato et al., 2014b).

Furthermore, we performed the MDTR on an unseen drug, Rosiglitazone, a drug that has a lack of medical reports concerning liver toxicity and was not utilized in the generation of dual-boundaries or the calculation of the ruler.

Case 3 We investigated the liver toxicity of Rosiglitazone, a thiazolidinedione drug used for the treatment of type 2 diabetes treatment but discontinued in many countries due to cardiovascular risks (Nissen and Wolski, 2007). While its counterpart Troglitazone has been withdrawn from the market due to severe hepatotoxicity (Lloyd et al., 2002; Kaplowitz, 2005), limited research exists on the hepatotoxic effects of Rosiglitazone. To address this gap, we employed the multi-dimensional ruler to evaluate its toxicity. In the PC3 cell line at the 24-h time point, Figure 4C demonstrates an increase in toxic mechanisms corresponding to higher drug doses. Specifically, significant variations were observed in Bile acids accumulation and Lipid metabolism in response to varying doses. These findings align with known issues associated with Rosiglitazone, such as disturbances in fatty acid and triglyceride metabolism (Gershell, 2005; Tan et al., 2005; Watkins et al., 2002), as well as intrahepatic cholestasis (Zhang et al., 2020).

Through comparative and case study results, we show that MDTR is an interpretable method that can capture both known and potentially mechanisms of toxicity, while exhibiting a dose-dependent distance to hepatotoxicity.

### 3.3 MoA interpretation: Oxidative stress as a case study

**FIGURE 5:**
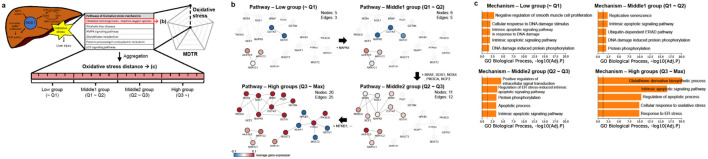
MoA of Oxidative stress **(A)** Conceptual illustration of liver injury caused by oxidative stress mechanism. **(B)** Co-expression network according to ROS pathway distance group. **(C)** GO biological process enrichment analysis according to oxidative stress mechanism distance group. The *x*-axis is represents -log (Adjusted *p*-value) and the dotted line represents -log (0.05).

Oxidative stress, one of the axis of MDTR, arises from an imbalance between oxygen-reactive species (ROS) generation and accumulation (Figure 5A, Jaeschke et al. (2002), Jaeschke et al. (2012)). The accumulation of ROS, triggered by factors such as AMP-activated protein kinase (AMPK) activation (Kang et al., 2016; Steinberg and Hardie, 2022) and an imbalance in glutathione (GSH) and glutathione disulfide (GSSG) levels (DeLeve and Kaplowitz, 1991; Yuan and Kaplowitz, 2009), promotes inflammation, disrupts cellular energy regulation, depletes antioxidants, impairs protein handling, and induces hepatocellular apoptosis, ultimately leading to liver damage.

To explore the biological implications of pathway-level distances within the mechanism, we focused on the Chemical carcinogenesis - ROS pathway (hsa05208, hereafter referred to as the ROS pathway), which is one of the six pathways chosen for the oxidative stress mechanism. We divided the samples evenly into four groups (Low, Middle1, Middle2, and High) based on each pathway distance. Then, we performed co-expression network analyses based on the expression values of samples within each group (Figure 5B). As a result, larger distances correspond to large co-expression network sizes, indicating that increased distance is associated with more interactions between genes and, simultaneously, a higher degree of gene dysregulation. For example, during the transition from the Low group to the Middle1 group, downregulation of MAPK8 (also known as JNK) may hinder the expression of the AP-1 transcription factor, potentially impacting the MAPK signaling pathway (Turner et al., 2014). Furthermore, as the network progressed from the Middle1 to Middle2 and High groups, upregulation of genes associated with PI3K signaling (e.g., PIK3CA or PIK3CD) and NADPH oxidase (e.g., NOX4) was observed, indicating ROS production (Koundouros and Poulogiannis, 2018). These findings validate the efficacy of the proposed pathway-level distance in capturing biological activity. Similar results were observed for the remaining pathways (Supplementary Figures S5-S9).

We next calculated the mechanism-level distance of oxidative stress as a weighted sum of the six related pathways, including the ROS pathway. To explore the association between oxidative stress signaling and the mechanism-level distance of samples, we performed GO enrichment analysis using commonly perturbed genes from the respective group. Figure 5C and Supplementary Figure S10 illustrate that as the distance increases, the GO terms related to oxidative stress, such as response to endoplasmic reticulum stress and cellular response to oxidative stress, exhibit more significant and abundant enrichment. These GO enrichment analysis results were consistently observed across all the mechanisms (Supplementary Figures S11-S14). Thus, our findings demonstrate that both pathway- and mechanism-level distance effectively reflect the activity level of the corresponding mechanism as the distance increases.

### 3.4 A web service for MDTR

Our model is implemented as a freely available website (http://biohealth.snu.ac.kr/software/MDTR). The website allows for our MDTR to be utilized from two perspectives (Supplementary Figure S15): (1) detection of hepatotoxicity from the user’s drug-induced transcriptome data, (2) investigation of hepatotoxicity through drug-induced transcriptome data in the LINCS database by selecting any combinations of drugs, tissues, and cell lines.

## 4 Conclusion

In this study, we introduced MDTR that quantifies the degree of liver toxicity in terms of five hepatotoxicity mechanisms by analyzing transcriptome data. An important contribution of this study is that it sought to translate transcriptome data to toxicity-related mechanisms between transcriptome data and DILI mechanisms. To understand the DILI mechanisms at the transcriptomic level, MDTR integrated the KEGG pathways, mapped the transcriptome data to the pathway-specific kernel space to measure the distance, and aggregated the pathway-level distance to measure the mechanism-level distance. MDTR explores complex genetic relationships in a non-linear RBF kernel space constructed from biological pathways, while at the same time having the explanatory power of DILI through a five-dimensional radar chart where integrated pathways are represented on one axis.

We showed that MDTR represents dose-dependent liver toxicity compared to existing models that measure distance or similarity of transcriptome data. In addition, through the case studies, we showed the ability of MDTR to interpret dose-dependent DILI not only for drugs with known liver toxicity but also for drugs with no reported liver toxicity. Furthermore, MDTR measures distances for different drug-treated environments, especially for different treatment doses, thereby capturing not only monotonic drug responses but also non-monotonic phenomena such as hormesis. Lastly, we provided a user-friendly and freely accessible website, enabling users to easily measure DILI in drug-induced transcriptome data. Therefore, MDTR serves as both an interpretable and computational method, addressing the limitations of existing studies that relied only on experimental and literature information to measure potential drug toxicity across various treatment environments.

While we have expanded the gene set based on the functional analysis of genes curated from the CTD, future studies may further enhance this gene set by including genes with correlated expression levels within the dataset. Moreover, although the current study involved a manual curation process to identify liver toxicity mechanisms and their associated biological pathways from the literature, we plan to conduct a more comprehensive analysis of liver toxicity by utilizing additional external resources, such as Ingenuity Pathway Analysis (IPA).

## Data Availability

The original contributions presented in the study are included in the article/Supplementary Material, further inquiries can be directed to the corresponding author.
