# Supplementary material for: MDTR: a knowledge-guided interpretable representation for quantifying liver toxicity at transcriptomic level
Source: Front Pharmacol. 2025 Jan 24;15:1398370. doi: 10.3389/fphar.2024.1398370 (PMC11802568; doi:10.3389/fphar.2024.1398370)
Supplement: Supplementary file 1 [file DataSheet1.pdf]

Supplementary Material

1 SUPPLEMENTARY METHODS

1.1 Drug hepatotoxicity information

Two publicly available databases, Drug Induced Liver Injury Rank (DILIrank) Chen et al. (2016) and LiverTox Hoofnagle (2013) are the main source for information on the hepatotoxicity of drugs categorized by medical literature or number of reported cases. The DILIrank Chen et al. (2016) provides a systemic classification of FDA-approved drugs based on their potential to cause DILI in humans. The evaluation schema used by DILIrank follows the Liver Toxicity Knowledge Base (LTKB) Benchmark Dataset Chen et al. (2011) conducted by FDA's National Center for Toxicological Research (NCTR). The drugs are classified into four categories based on their potential to cause drug-induced liver injury (DILI): most concern, less concern, no concern, and ambiguous concern. For this study, drugs categorized as most-DILI concern in the DILIrank database were considered as hepatotoxic drugs, while drugs categorized as no-DILI concern were considered as non-hepatotoxic drugs.

LiverTox Hoofnagle (2013) provides up-to-date information on the diagnosis, cause, frequency, clinical characteristics, and management of liver damage caused by prescription and generic, approved and withdrawn drugs. Using the Drug-Induced Liver Injury Network (DILIN) Fontana et al. (2009), LiverTox categorizes drugs into five likelihood scores from A to E, indicating the extent of reported liver injury cases. For this study, drugs categorized as A and B in the LiverTox database were considered as hepatotoxic drugs, while drugs categorized as D and E were considered as non-hepatotoxic drugs.

In this study, a total of 220 drugs categorized as hepatotoxic and 402 drugs categorized as non-hepatotoxic were used, using information from both DILIrank and LiverTox. The statistics and data source of drug hepatotoxicity are in Supplementary Table S10.

1.2 Drug-treated transcriptome data preparation

To analyze toxic signatures at the transcriptome level, we require experimentally measured gene expression data that has been perturbed by drug treatment. In this study, we used publicly available data deposited in the LINCS L1000 database for comprehensive gene expression changes via drug treatment on various cell lines in various time points with different amount of doses. We obtained the LINCS L1000 data from the Gene Expression Omnibus (GEO) using the accession number GSE92742. We utilized the level 5 expression samples that provide robust differential expressions considering control and replication. To focus on the hepatotoxicity of drugs, we utilized a subset of drug-treated transcriptome data in LINCS for which the drug-treated samples were curated in the "Drug hepatotoxicity information" section. DMSO-treated samples were also used to consider the state of cells before drug treatment. As a result, we used 20,529 drug-treated samples consisting of 6,405 toxic drug-treated samples and 11,333 non-toxic drug-treated samples, and 2,791 DMSO-treated samples. These samples were generated at 70 cell lines, three time points (6, 24, and 48 hours), and 84 dose points (ranging from 0.0012207 to 100 μM).

1.2.1 Gene selection process

LINCS perturbation samples contain experimentally measured expression levels of 978 landmark genes and the inferred gene expressions using these landmark genes. Among the LINCS 10,174 best inference genes (BINGs) with significant inference accuracy Subramanian et al. (2017), we selected genes related to

drug-induced liver injury. To curate the informative genes, we first collected 447 genes specifically reported to be directly related as markers or mechanisms known to be associated with 'Chemical and Drug Induced Liver Injury' based on 3,331 literatures in the disease category of the Comparative Toxicogenomics Database (CTD) Davis et al. (2021). Another alternative database for obtaining 'disease-gene' information is DisGeNET. However, DisGeNET is a commercial resource, which limits its accessibility. For comparison, we accessed two specific disease terms from DisGeNET through Enrichr: 'Drug-Induced Acute Liver Injury' with 324 genes and 'Drug Toxicity' with 82 genes. We compared the gene sets from CTD and DisGeNET to assess their robustness. As a result, we found that 'Drug-Induced Acute Liver Injury' in DisGeNET overlapped significantly with the CTD 'Chemical and Drug Induced Liver Injury' category, with 97.5% of the genes included in CTD (Table S14). On the other hand, 'Drug Toxicity' being a broader category than DILI, had less overlap. However, when compared with the expanded CTD+GO gene set obtained using GO terms, there was an 87.8% overlap, indicating that our selected gene set is comprehensive and robust.

We then performed an enrichment experiment using the 447 genes, which resulted in 7,167 genes with enriched Gene Ontology (GO) biological process (BP) terms (adjusted p-value < 0.05, Table S11 for GO BP, additionally Table S12 for GO Cellular Component, and Table S13 for GO Molecular Function). We conducted comparative experiments using three additional functional analysis approaches: KEGG, Reactome, and WikiPathways, alongside GO, starting with the 447 CTD genes. The results showed that the GO analysis provided the most extensive expansion of the gene set (Table S15). Approximately 70% of the genes collected from the other methods (KEGG, Reactome, WikiPathways) were included within the GO-based gene set. It is important to recognize that a large number of genes is not necessarily advantageous, as it could lead to an increase in false positives. However, since this step is the starting point for collecting genes related to liver toxicity, beginning with a limited gene set, as the reviewer suggested, would narrow the scope of the downstream analysis. Therefore, in this initial phase, we aimed to include as many reasonable genes as possible.

Finally, we selected 4,693 genes that overlapped with the 10,174 BINGs. The 447 CTD genes, 7,167 CTD-GO genes, and 4,693 genes used in the analysis are presented in Table S16.

1.3 Pathway selection process

To curate the KEGG pathways relevant to each mechanism, we utilized the KEGG pathway search function (available at (https://www.genome.jp/kegg/pathway.html) with search terms specific to each mechanism, as outlined in the 'Search Term' column in Table S17. For example, the 'Immunological response' mechanism was queried using the term 'inflammation,' while other mechanisms had their own respective search terms. The initial search resulted in a larger pool of pathways, as indicated in the '# of searched pathways' column in Table S17. We then manually curated these search results to ensure that the pathways included were specifically relevant to the mechanisms involved in drug-induced liver toxicity (DILI). During this curation process, some pathways that were initially identified through the search—such as Systemic Lupus Erythematosus (SLE) and Rheumatoid Arthritis (RA)—were excluded because they have limited direct relevance to drug-induced liver injury, despite their broader immunological implications. This led to a reduction in the number of immune-related pathways to only three, as shown in the 'Number of curated DILI pathways' column in Table S17.

1.4 Simulation data with toxic signatures

Classifying samples with toxicity signatures as boundary criteria is necessary to establish well-defined toxicity boundaries. To identify the effectiveness of the two boundaries configured with the Dual-SVDD, we generated simulation data with toxicity signatures. To ensure a fair comparison, we used PT samples that were commonly captured by Dual-SVDD and single boundary methods. We acquired DMSO-treated samples that underwent the same treatment conditions (cell line, time, and dosage), which were denoted as $DMSO^{PT}$. We then created the simulation data $\tilde{X}$ by mixing the PT samples and corresponding $DMSO^{PT}$, as follows:

$$\tilde{X} = \alpha * PT + (1 - \alpha) * DMSO^{PT} \tag{S1}$$

where PT represents the gene expression quantity of a single potential toxic sample, and $DMSO^{PT}$ represents a DMSO-treated transcriptome sample with the same conditions. The mixing ratio, α, ranged from 0.0 to 1.0 in increments of 0.1.

1.5 Cross-check evaluation of one-dimensional ruler

To show the robustness of our distance measurement unaffected by the specific characteristics of the transcriptomic data, we conducted an additional cross-check evaluation of the distance on the partitioned LINCS dataset. For convenience of comparison, we considered all genes in LINCS dataset as a single mechanism composed of a single pathway, and computed the mechanism-level distance as a one-dimensional ruler. Specifically, we first divided the dataset evenly into k partitions. Next, we created k one-dimensional rulers using only the data belonging to each partition. Finally, we calculated pairwise similarities by measuring the distances between all partitions using distances measured by k one-dimensional rulers.

1.6 Comparison of dose-dependent liver toxicity

In this study, we compared the MDTR distance with three different methods, (1) raw LINCS gene expression, (2) Transcriptomic Signature Distance (TSD) Manatakis et al. (2020), and (3) Pathway Activity Score Learning (PASL) Karagiannaki et al. (2023), to assess the correlation between sample distance and drug dose.

Raw LINCS gene expression: First of all, we compared MDTR with raw gene expression from drug-induced transcriptome data in LINCS. This comparison will allow us to observe the differences without prior knowledge. Similar to MDTR, only the expression values of 4693 genes out of 10174 were utilized. Distances between samples were calculated based on the average expression value across the entire dataset, employing three mathematical distances: cosine, Euclidean, and Mahalanobis.

Transcriptomic Signature Distance (TSD): TSD, introduced by Manatakis et al. (2020), is an information-theoretic distance measurement based on human organ characteristic gene sets. TSD measures the distance between samples using the Jensen-Shannon Divergence value and Rankings Correlation Coefficient value for a given gene set. When calculating the Jensen-Shannon Divergence value, positive gene expression values are required, so we scaled LINCS gene expression data using the Min-Max scaler. And then, the distance of each sample was calculated through TSD based on the overall sample average.

Pathway Activity Score Learning (PASL): PASL, proposed by Karagiannaki et al. (2023), transforms high-dimensional transcriptomic data into pathway activity scores through PCA-based dimension reduction, offering a more focused and interpretable view of cellular responses. PASL requires a gene membership

matrix, denoting the presence or absence of genes in the specified gene sets. Accordingly, we created a gene membership matrix based on the gene set of 25 KEGG pathways used in MDTR. With activity scores obtained with PASL, sample distances were calculated using three mathematical distances (cosine, Euclidean, and Mahalanobis) based on the overall average of activity scores.

2 SUPPLEMENTARY TABLES AND FIGURES

2.1 Figures

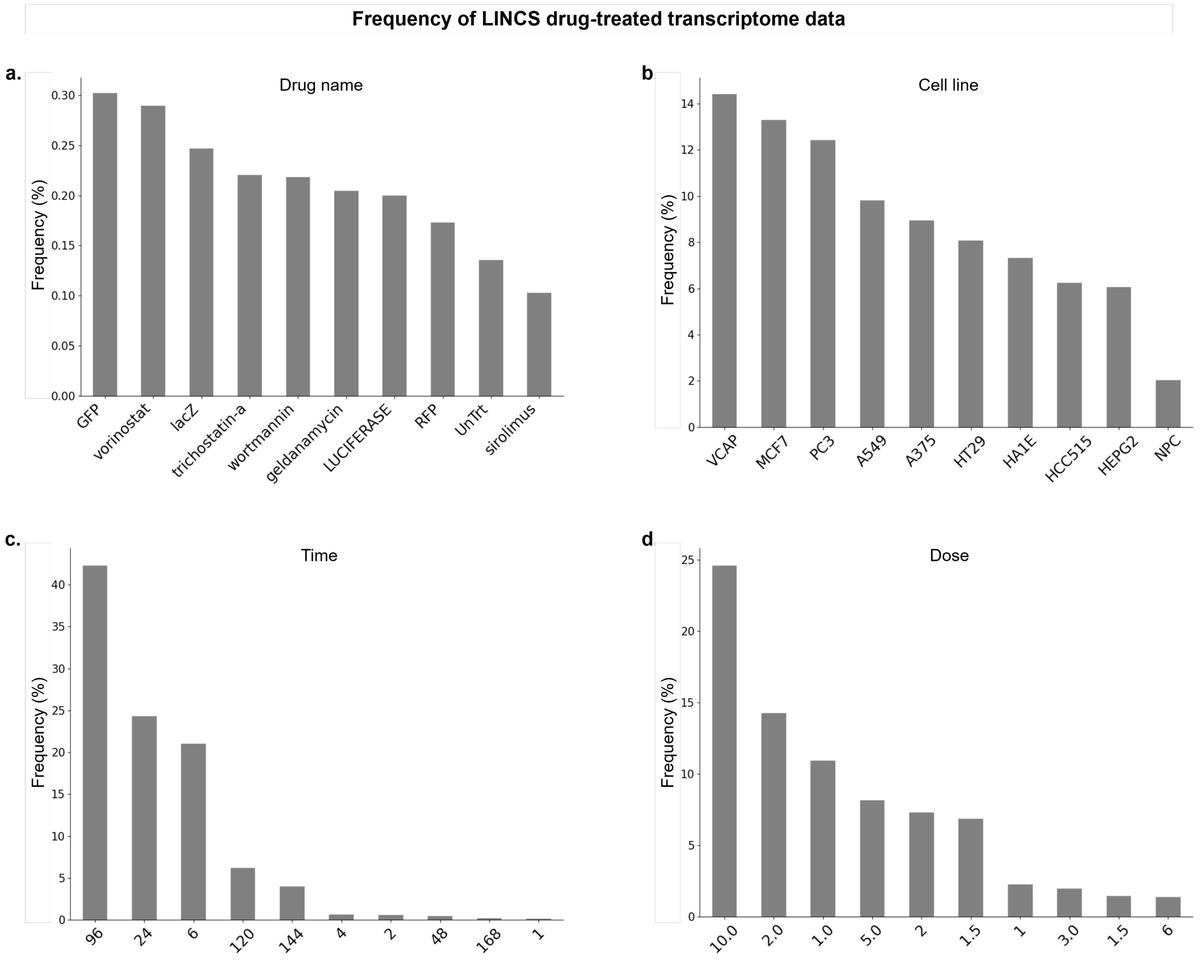

Figure S1: **Information about the LINCS dataset.** The bar plots show the frequency of (a) drugs, (b) cell lines, (c) time, and (d) dose points in the drug-treated samples provided by LINCS, excluding samples treated with DMSO and empty vector.

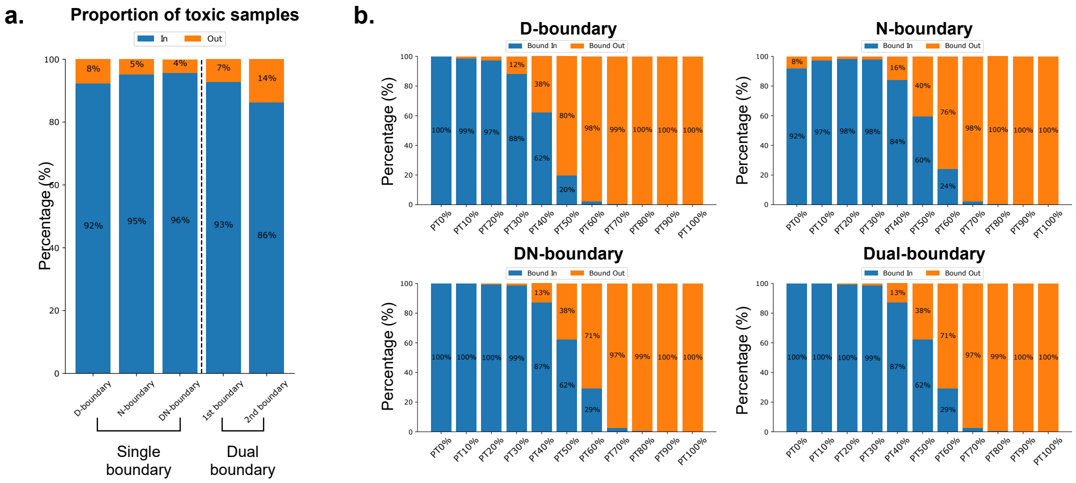

Figure S2: **Comparison the single boundary methods and Dual-SVDD.** For each boundary, (a) the stacked bars show the percentage of PT samples inside (blue) and outside (orange) the boundary, and (e) the plots show the percentage of simulation data inside (blue) and outside (orange for the first, green for the second) the boundary. D-boundary: boundary generated using DMSO samples. N-boundary: boundary generated using non-toxic samples. DN-boundary: boundary generated using DMSO and non-toxic samples.

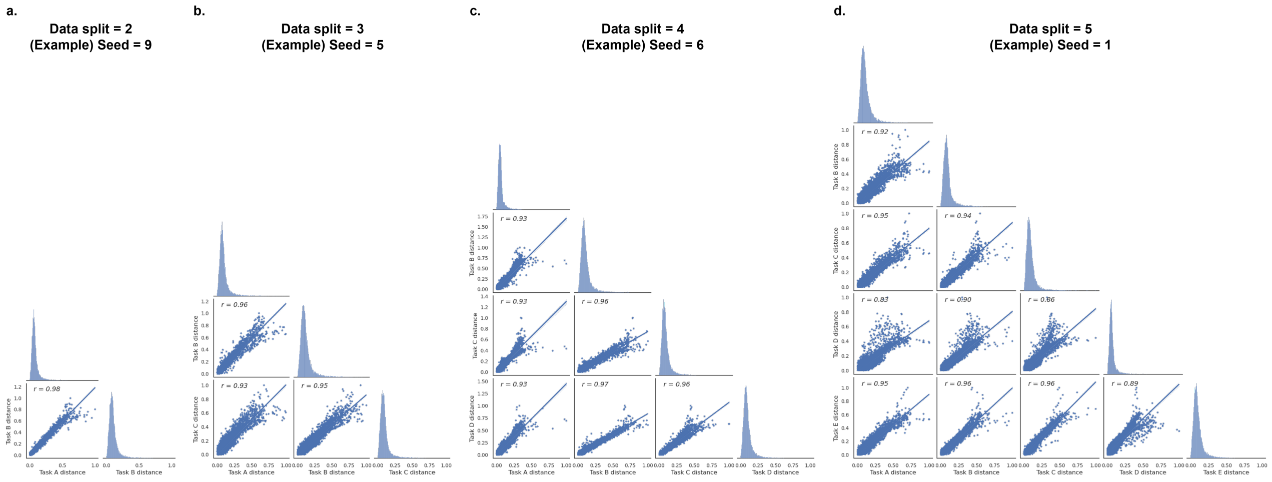

Figure S3: **Examples of pairwise relationship of distances.** When data were divided into (a) two, (b) three, (c) four, and (d) five equal datasets, the scatter plots show pairwise comparisons of distances within each dataset and Pearson's correlation coefficient.

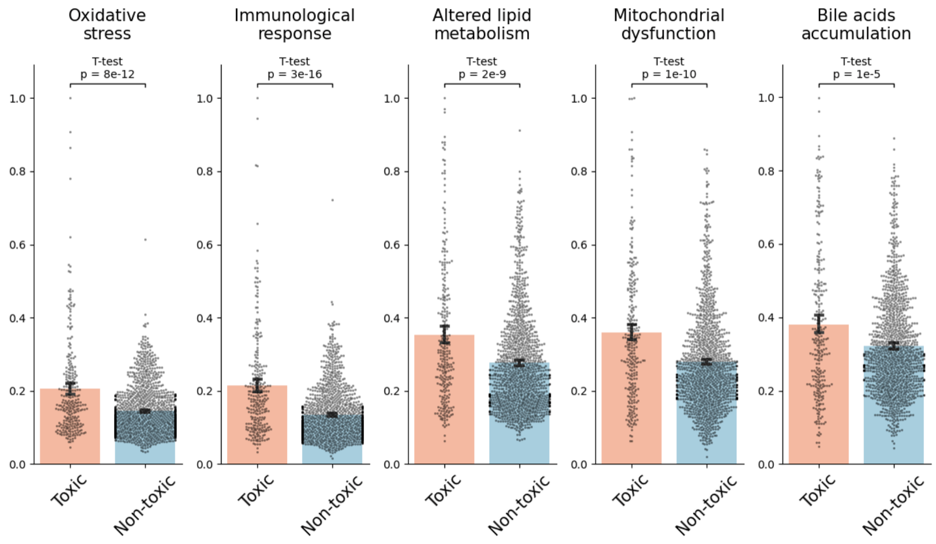

Figure S4: **Comparison of distances between toxic and non-toxic samples on the entire cell lines.** The bar graph shows the distance between 274 toxic drug samples (red) and 1,236 non-toxic drug (blue) for the 61 cell lines in LINCS.

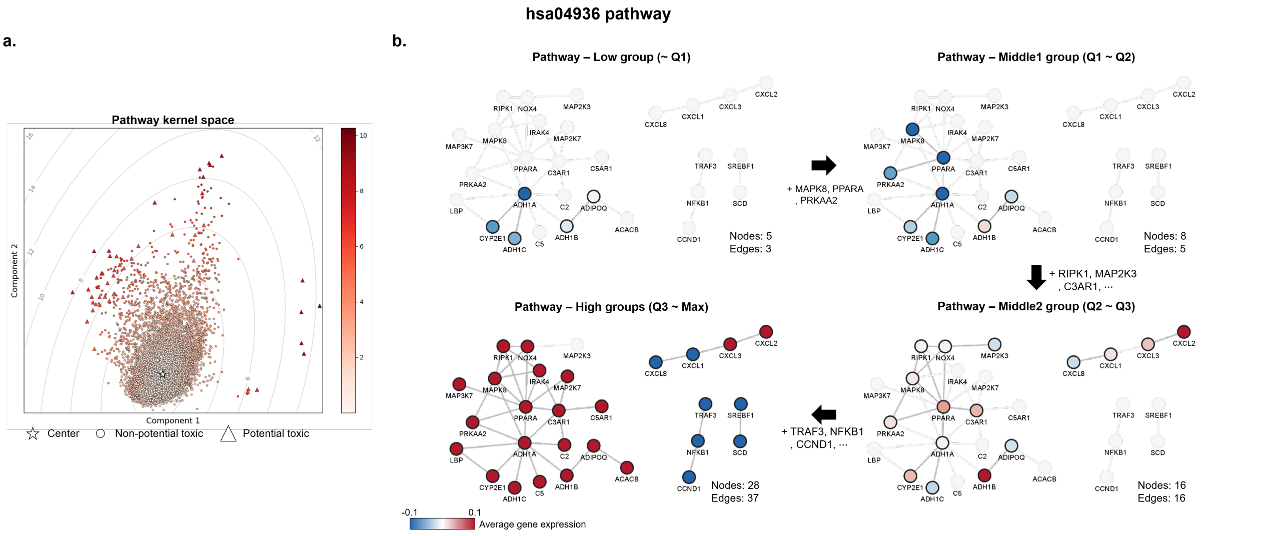

Figure S5: **Oxidative stress - Alcoholic liver disease pathway distance results.** (a) The kernel space of the Alcoholic liver disease pathway. Each point represents a drug-treated transcriptome sample (circle point: non-PT and triangle: PT sample) and color of the points becomes redder as the distance from the center (star point) increase. (b) Co-expression networks in the distance groups. The co-expression network is constructed based on the gene-gene Pearson's correlation coefficient within each distance group (correlation cutoff = 0.5).

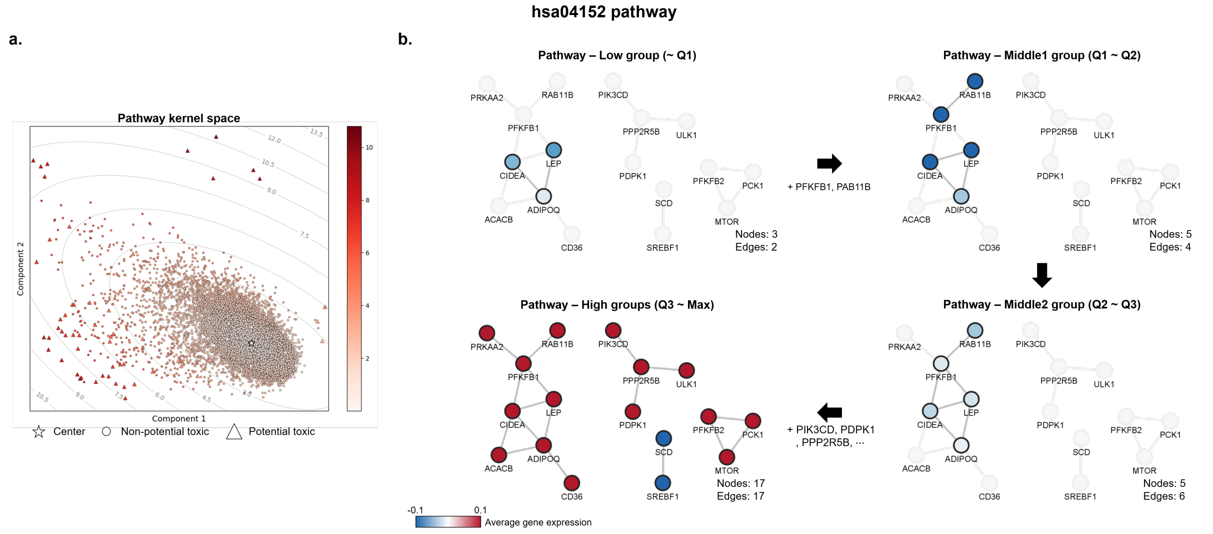

Figure S6: **Oxidative stress - AMPK signaling pathway distance results.** (a) The kernel space of the AMPK signaling pathway. Each point represents a drug-treated transcriptome sample (circle point: non-PT and triangle: PT sample) and color of the points becomes redder as the distance from the center (star point) increase. (b) Co-expression networks in the distance groups. The co-expression network is constructed based on the gene-gene Pearson's correlation coefficient within each distance group (correlation cutoff = 0.5).

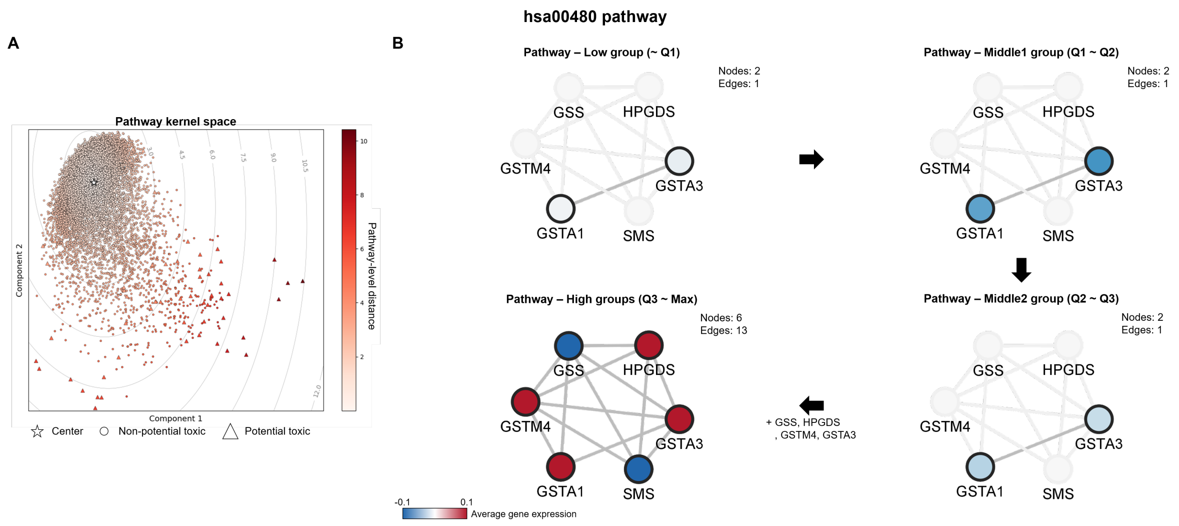

Figure S7: **Oxidative stress - Glutathione metabolism distance results.** (a) The kernel space of the Glutathione metabolism pathway. Each point represents a drug-treated transcriptome sample (circle point: non-PT and triangle: PT sample) and color of the points becomes redder as the distance from the center (star point) increase. (b) Co-expression networks in the distance groups. The co-expression network is constructed based on the gene-gene Pearson's correlation coefficient within each distance group (correlation cutoff = 0.5).

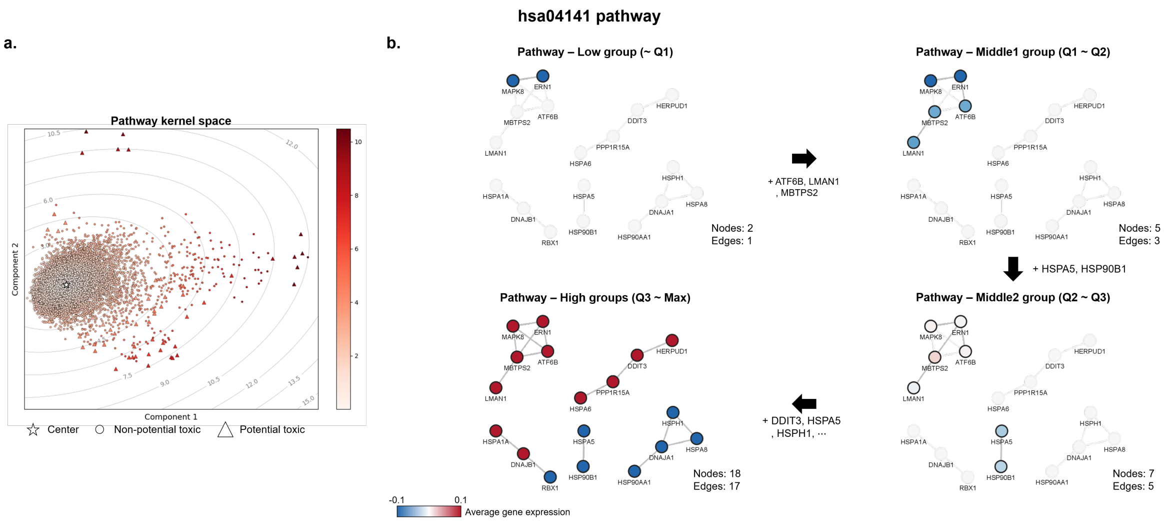

Figure S8: **Oxidative stress - Protein processing in ER pathway distance results.** (a) The kernel space of the Protein processing in ER pathway. Each point represents a drug-treated transcriptome sample (circle point: non-PT and triangle: PT sample) and color of the points becomes redder as the distance from the center (star point) increase. (b) Co-expression networks in the distance groups. The co-expression network is constructed based on the gene-gene Pearson's correlation coefficient within each distance group (correlation cutoff = 0.5). ER: Endoplasmic reticulum.

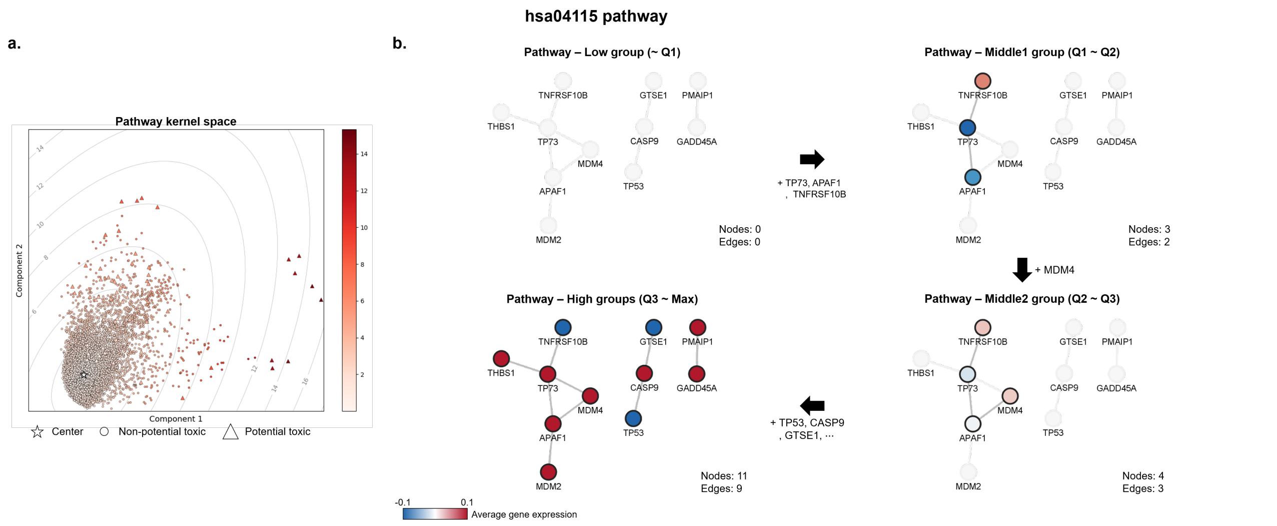

Figure S9: **Oxidative stress - p53 signaling pathway distance results.** (a) The kernel space of the p53 signaling pathway. Each point represents a drug-treated transcriptome sample (circle point: non-PT and triangle: PT sample) and color of the points becomes redder as the distance from the center (star point) increase. (b) Co-expression networks in the distance groups. The co-expression network is constructed based on the gene-gene Pearson's correlation coefficient within each distance group (correlation cutoff = 0.5).

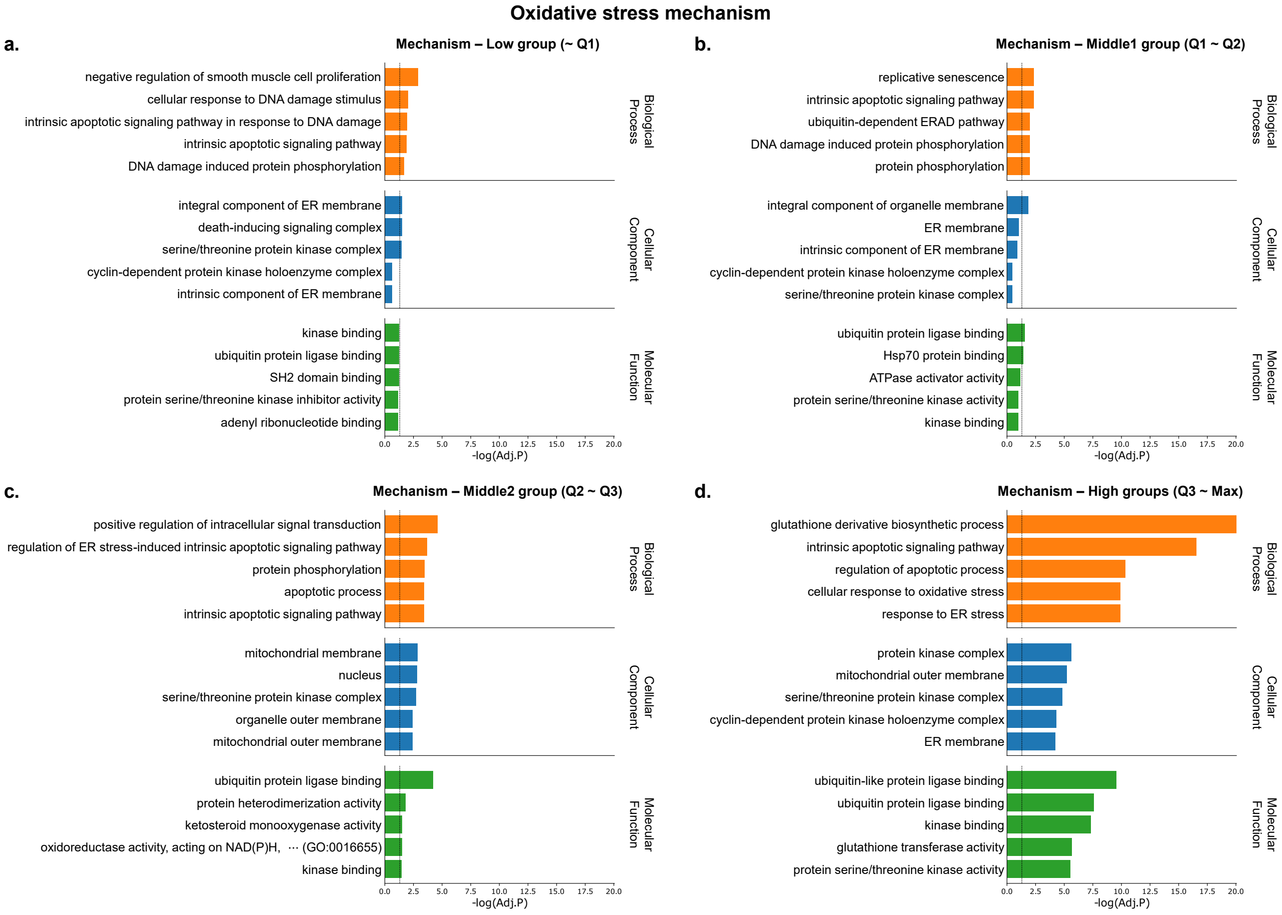

Figure S10: **GO enrichment analysis for Oxidative stress.** (a-d) GO enrichment analysis was conducted for each group (a) Low, (b) Middle1, (c) Middle2, and (d) High. The analysis used common DEGs within the group. The bar plots show the top 5 GO terms with low Adjusted p-value in the Biological Process, Cellular Component, and Molecular Function categories. The dashed line represents an Adjusted p-value of 0.05. GO: Gene Ontology, ROS: reactive oxygen species, ER: Endoplasmic reticulum.

2.2 Tables

Table S1. List of pathways associated with Oxidative stress mechanism. *ROS: Reactive oxygen species, AMPK: Adenosine monophosphate-activated protein kinase, mER: Endoplasmic reticulum, JNK: c-Jun N-terminal Kinase, APAP: Acetaminophen.

KEGG	Pathway	Function	Reference
hsa05208	Chemical carcinogenesis - ROS	Chemical carcinogens induce ROS through mitochondrial stress and immune responses	Jaeschke et al. (2002, 2012)
hsa04936	Alcoholic liver disease	Chronic alcohol consumption elevates ROS, causing liver injury	O'shea et al. (2010); Seitz et al. (2018)
hsa04152	AMPK signaling pathway	Oxidative stress inhibits AMPK, impairing liver protection	Kang et al. (2016); Steinberg and Hardie (2022)
hsa00480	Glutathione metabolism	Depleted glutathione weakens liver's ROS defense	DeLeve and Kaplowitz (1991); Yuan and Kaplowitz (2009)
hsa04141	Protein processing in ER	ROS and ER stress are linked; disrupted folding environment triggers oxidative stress	Malhi and Kaufman (2011)
hsa04115	p53 signaling pathway	p53 inhibits JNK activation, protecting against APAP-induced oxidative stress	Kurinna et al. (2013); Ally et al. (2017)

Table S2. List of pathways associated with Immunological response mechanism.

KEGG	Pathway	Function	Reference
hsa04064	-kappa B signaling pathway	-kB activation links to inflammatory diseases	Liu et al. (2017)
hsa04936	Alcoholic liver disease	Ethanol's MAA-adduct studied for alcoholic liver disease onset	Duryee et al. (2007)
hsa04932	Non-alcoholic fatty liver disease	Immune response drives NAFLD initiation and progression	Paquissi (2016)

Table S3. List of pathways associated with Altered lipid metabolism mechanism.

KEGG	Pathway	Function	Reference
hsa00061<br>hsa00062<br>hsa00071	Fatty acid biosynthesis<br>Fatty acid elongation<br>Fatty acid degradation	Drug-induced metabolic alterations in the liver lead to triglyceride accumulation and liver injury	Alves-Bezerra and Cohen (2017)
hsa00100<br>hsa00140	Steroid biosynthesis<br>Steroid hormone biosynthesis	Dysregulation of steroid metabolism in the liver due to drugs disrupts hormone production and elimination	Charni-Natan et al. (2019)
hsa00561<br>hsa00564<br>hsa00565<br>hsa00600	Glycerolipid metabolism<br>Glycerophospholipid metabolism<br>Ether lipid metabolism<br>Sphingolipid metabolism	Liver plays a key role in glycerolipid metabolism, particularly in the GL/FFA cycle	Prentki and Madiraju (2008)
hsa00590<br>hsa00591<br>hsa00592<br>hsa01040	Arachidonic acid metabolism<br>Linoleic acid metabolism<br>alpha-Linolenic acid metabolism<br>Biosynthesis of unsaturated fatty acids	Imbalanced PUFAs, especially reduced omega-3 PUFAs, contribute to liver injury through inflammation and oxidative stress	Lee et al. (2020)

Table S4. List of pathways associated with Mitochondrial dysfunction mechanism.

KEGG	Pathway	Function	Reference
hsa00190	Oxidative phosphorylation	Hepatocyte oxidative phosphorylation impairment causes ATP depletion, calcium accumulation, necrosis	Pessayre et al. (1999); Begriche et al. (2011)
hsa00020	Citrate cycle (TCA cycle)	TCA cycle impairment leads to toxic environments, disrupting energy balance.	Begriche et al. (2011)

Table S5. List of pathways associated with Bile acids accumulation mechanism.

KEGG	Pathway	Function	Reference
hsa00120	Primary bile acid biosynthesis	Drugs can induce bile acid synthesis, increasing toxic bile acids and causing hepatocellular damage	Schadt et al. (2016)
hsa04976	Bile secretion	Bile acids are secreted into bile by BSEP; drug-mediated BSEP impairment leads to intracellular bile acid accumulation and hepatocellular damage	Woodhead et al. (2014)

Table S6. List of potential toxic samples.

Drug Name	Cell line	Time (hour)	Dose (μM)
Amodiaquine	HEPG2	6	10
	NEU	24	10
Celecoxib	A549	24	10
Dactinomycin	A549	6	10
	HA1E	24	10
	HCC515	24	10
	MCF7	6	10
	VCAP	24	10
Diflunisal	HA1E	6	10
Doxorubicin	A375	24	0.04
			0.12
			0.37
			1.11
			3.33
	A549	24	0.37
			1.11
			3.33
	HCC515	24	10
			10
	MCF7	6	10
		24	10
	PC3	24	10
	VCAP	6	10
Etodolac	HA1E	6	10
Febuxostat	HEPG2	6	10
Fluorouracil	MCF7	24	10
Flutamide	A375	6	10
	SNUC5	6	28.96
Gemcitabine	NCIH596	6	0.08
Haloperidol	HT29	6	10
Indomethacin	HA1E	6	10
Mitomycin	HA1E	24	10
Mitomycin	SKB	24	10
Quinine	HCC15	6	10
	JHUEM2	6	10
	SNUC5	6	10
	SW948	6	10
Stavudine	PC3	6	10
Sulfasalazine	PC3	6	10
Tamoxifen	HA1E	24	10
	HEPG2	24	10
Temozolomide	NEU	24	10
	PC3	24	0.3704
Terbinafine	MCF7	6	10
Thioridazine	VCAP	6	10
Troglitazone	A375	24	3.33333
	A549	24	3.33333
	HT29	24	3.33333
	MCF7	24	3.33333
			3.33333
	NEU	24	3.33333
			3.33333
	PC3	6	2
		24	0.08
			3.33333
	VCAP	6	0.08
			0.08
			2
		24	0.08
			0.08
			2
			2
Verapamil	T3M10	6	10
Voriconazole	HA1E	6	10

Table S7. Performance comparison for dose-distance relationship. *Bold values indicate the best results.

A total of 533 combinations of 74 drugs with three or more drug administration points.

Method	Positive ratio (Number of positive samples)
**MDTR**	**0.71 (381)**
Raw + Cosine	0.62 (328)
Raw + Euclidean	0.68 (363)
Raw + Mahalanobis	0.66 (354)
TSD	0.70 (372)
PASL + Cosine	0.51 (271)
PASL + Euclidean	0.62 (331)
PASL + Mahalanobis	0.71 (377)

A total of 520 combinations of 73 drugs with four or more drug administration points.

Method	Positive ratio (Number of positive samples)
**MDTR**	**0.72 (375)**
Raw + Cosine	0.62 (322)
Raw + Euclidean	0.69 (357)
Raw + Mahalanobis	0.67 (347)
TSD	0.70 (366)
PASL + Cosine	0.51 (264)
PASL + Euclidean	0.71 (368)
PASL + Mahalanobis	0.71 (377)

Table S8. Spearman correlation between Doxorubicin dose and distance in each cell line over time.

Drug	Cell line	Time (hour)	Number of samples	Number of dose points	Correlation (ρ)
Doxorubicin	MCF7	6	14	9	0.928743
	MCF7	24	18	12	0.899851
	PC3	24	10	6	0.898646
	HEPG2	24	6	6	0.885714
	HT29	24	6	6	0.828571
	A549	6	12	9	0.815076
	A549	24	18	12	0.76623
	HCC515	24	8	6	0.707528
	HA1E	24	6	6	0.428571
	A375	24	6	6	-0.028571

Table S9. Spearman correlation between Mitoxantrone dose and distance in each cell line over time.

Drug	Cell line	Time (hour)	Number of samples	Number of dose points	Correlation (ρ)
Mitoxantrone	A549	24	10	9	0.954412
	A549	6	11	9	0.954169
	MCF7	24	16	12	0.904578
	PC3	6	6	4	0.880406
	MCF7	6	16	12	0.874724
	PC3	24	7	4	0.866921

Table S10. The drug labels for hepatotoxicity.

	Data source				
Drug hepatotoxicity lable	DILIrank Concern	Count	LiverTox Likelihood	count	Total
Toxic	Most	134	A<br>B	66<br>88	220
Non-toxic	No	174	D<br>E	99<br>195	402

Table S11. GO enrichment results for 'Chemical and Drug Induced Liver Injury' in the CTD database.

GO Biological Process Term	Adjusted p-value	Combined score
long-chain fatty acid biosynthetic process (GO:0042759)	6.50E-08	639.7168
glutathione metabolic process (GO:0006749)	1.55E-07	401.1558
long-chain fatty acid metabolic process (GO:0001676)	2.45E-07	216.5417
glutathione derivative biosynthetic process (GO:1901687)	2.45E-07	666.1778
glutathione derivative metabolic process (GO:1901685)	2.45E-07	666.1778
positive regulation of cytokine production (GO:0001819)	2.45E-07	93.88745
cytokine-mediated signaling pathway (GO:0019221)	2.45E-07	70.64053
regulation of neuroinflammatory response (GO:0150077)	1.14E-06	633.8649
epoxygenase P450 pathway (GO:0019373)	1.14E-06	633.8649
exogenous drug catabolic process (GO:0042738)	3.88E-06	462.6039

REFERENCES

Ally, A., Balasundaram, M., Carlsen, R., Chuah, E., Clarke, A., Dhalla, N., et al. (2017). Comprehensive and integrative genomic characterization of hepatocellular carcinoma. *Cell* 169, 1327–1341

Alves-Bezerra, M. and Cohen, D. E. (2017). Triglyceride metabolism in the liver. *Comprehensive Physiology* 8, 1

Begriche, K., Massart, J., Robin, M.-A., Borgne-Sanchez, A., and Fromenty, B. (2011). Drug-induced toxicity on mitochondria and lipid metabolism: mechanistic diversity and deleterious consequences for the liver. *Journal of hepatology* 54, 773–794

Charni-Natan, M., Aloni-Grinstein, R., Osher, E., and Rotter, V. (2019). Liver and steroid hormones—can a touch of p53 make a difference? *Frontiers in endocrinology* 10, 374

Chen, M., Suzuki, A., Thakkar, S., Yu, K., Hu, C., and Tong, W. (2016). Dilirank: the largest reference drug list ranked by the risk for developing drug-induced liver injury in humans. *Drug Discov Today* 21, 648–653

Chen, M., Vijay, V., Shi, Q., Liu, Z., Fang, H., and Tong, W. (2011). Fda-approved drug labeling for the study of drug-induced liver injury. *Drug discovery today* 16, 697–703

Davis, A. P., Grondin, C. J., Johnson, R. J., Sciaky, D., Wiegers, J., Wiegers, T. C., et al. (2021). Comparative toxicogenomics database (ctd): update 2021. *Nucleic acids research* 49, D1138–D1143

DeLeve, L. D. and Kaplowitz, N. (1991). Glutathione metabolism and its role in hepatotoxicity. *Pharmacology & therapeutics* 52, 287–305

Duryee, M. J., Klassen, L. W., and Thiele, G. M. (2007). Immunological response in alcoholic liver disease. *World journal of gastroenterology: WJG* 13, 4938

Fontana, R. J., Watkins, P. B., Bonkovsky, H. L., Chalasani, N., Davern, T., Serrano, J., et al. (2009). Drug-induced liver injury network (dilin) prospective study: rationale, design and conduct. *Drug safety* 32, 55–68

Hoofnagle, J. H. (2013). Livertox: a website on drug-induced liver injury. In *Drug-Induced Liver Disease* (Elsevier). 725–732

Jaeschke, H., Gores, G. J., Cederbaum, A. I., Hinson, J. A., Pessayre, D., and Lemasters, J. J. (2002). Mechanisms of hepatotoxicity. *Toxicological sciences* 65, 166–176

Jaeschke, H., McGill, M. R., and Ramachandran, A. (2012). Oxidant stress, mitochondria, and cell death mechanisms in drug-induced liver injury: lessons learned from acetaminophen hepatotoxicity. *Drug metabolism reviews* 44, 88–106

Kang, S. W. S., Haydar, G., Taniane, C., Farrell, G., Arias, I. M., Lippincott-Schwartz, J., et al. (2016). Ampk activation prevents and reverses drug-induced mitochondrial and hepatocyte injury by promoting mitochondrial fusion and function. *PloS one* 11, e0165638

Karagiannaki, I., Gourlia, K., Lagani, V., Pantazis, Y., and Tsamardinos, I. (2023). Learning biologically-interpretable latent representations for gene expression data: pathway activity score learning algorithm. *Machine Learning* 112, 4257–4287

Kurinna, S., Stratton, S. A., Coban, Z., Schumacher, J. M., Grompe, M., Duncan, A. W., et al. (2013). p53 regulates a mitotic transcription program and determines ploidy in normal mouse liver. *Hepatology* 57, 2004–2013

Lee, C.-H., Fu, Y., Yang, S.-J., and Chi, C.-C. (2020). Effects of omega-3 polyunsaturated fatty acid supplementation on non-alcoholic fatty liver: a systematic review and meta-analysis. *Nutrients* 12, 2769

Liu, T., Zhang, L., Joo, D., and Sun, S.-C. (2017). Nf-κb signaling in inflammation. *Signal transduction and targeted therapy* 2, 1–9

Malhi, H. and Kaufman, R. J. (2011). Endoplasmic reticulum stress in liver disease. *Journal of hepatology* 54, 795–809

Manatakis, D. V., VanDevender, A., and Manolakos, E. S. (2020). An information-theoretic approach for measuring the distance of organ tissue samples using their transcriptomic signatures. *Bioinformatics* 36, 5194–5204

O'shea, R. S., Dasarathy, S., McCullough, A. J., of the American Association for the Study of Liver Diseases, P. G. C., of the American College of Gastroenterology, P. P. C., et al. (2010). Alcoholic liver disease. *Hepatology* 51, 307–328

Paquissi, F. C. (2016). Immune imbalances in non-alcoholic fatty liver disease: from general biomarkers and neutrophils to interleukin-17 axis activation and new therapeutic targets. *Frontiers in immunology* 7, 490

Pessayre, D., Mansouri, A., Haouzi, D., and Fromenty, B. (1999). Hepatotoxicity due to mitochondrial dysfunction. *Cell biology and toxicology* 15, 367–373

Prentki, M. and Madiraju, S. M. (2008). Glycerolipid metabolism and signaling in health and disease. *Endocrine reviews* 29, 647–676

Schadt, H. S., Wolf, A., Pognan, F., Chibout, S.-D., Merz, M., and Kullak-Ublick, G. A. (2016). Bile acids in drug induced liver injury: Key players and surrogate markers. *Clinics and research in hepatology and gastroenterology* 40, 257–266

Seitz, H. K., Bataller, R., Cortez-Pinto, H., Gao, B., Gual, A., Lackner, C., et al. (2018). Alcoholic liver disease. *Nature reviews Disease primers* 4, 16

Steinberg, G. R. and Hardie, D. G. (2022). New insights into activation and function of the ampk. *Nature Reviews Molecular Cell Biology* , 1–18

Subramanian, A., Narayan, R., Corsello, S. M., Peck, D. D., Natoli, T. E., Lu, X., et al. (2017). A next generation connectivity map: L1000 platform and the first 1,000,000 profiles. *Cell* 171, 1437–1452

Woodhead, J. L., Yang, K., Siler, S. Q., Watkins, P. B., Brouwer, K. L., Barton, H. A., et al. (2014). Exploring bsep inhibition-mediated toxicity with a mechanistic model of drug-induced liver injury. *Frontiers in pharmacology* 5, 240

Yuan, L. and Kaplowitz, N. (2009). Glutathione in liver diseases and hepatotoxicity. *Molecular aspects of medicine* 30, 29–41
